# Acute Chest Syndrome, Avascular Necrosis of Femur, and Pulmonary Embolism All at Once: An Unexpected Encounter in the First-Ever Admission of a Sickle Cell Patient

**DOI:** 10.7759/cureus.17656

**Published:** 2021-09-01

**Authors:** Akhilesh Annadatha, Dhruv Talwar, Sourya Acharya, Sunil Kumar, Vivek Lahane

**Affiliations:** 1 Department of Medicine, Jawaharlal Nehru Medical College, Datta Meghe Institute of Medical Sciences (Deemed University), Wardha, IND

**Keywords:** sickle cell disease, avascular necrosis of femur, acute chest syndrome, pulmonary embolism, sickle cell crisis

## Abstract

Acute chest syndrome (ACS) is defined as the radiological appearance of pulmonary infiltrates with fever or respiratory symptoms like chest pain, breathlessness, and cough in a patient with sickle cell disease (SCD). It is also a very common cause of mortality in sickle cell patients, if not identified in early stages and treated aggressively. Radiological image is similar to bacterial pneumonia, so sickle cell disease with a radiological picture similar to pneumonia and associated respiratory symptoms is known as acute chest syndrome. Pneumonia and infarction have been implicated in pathogenesis. The reason for the appearance of acute chest syndrome in patients with SCD is not established but some triggers like sepsis, presence of vaso-occlusive crises have been noted. When there is a block in the blood supply to the bone, patients with sickle cell disease may also develop avascular necrosis of the neck of the femur causing narrowing of joint and collapse of the bone. Patients with sickle cell disease have a baseline hypercoagulable state thereby predisposing the patient to develop deep vein thrombosis and pulmonary embolism. Here, we present a case of a 25-year-old SCD patient with a fairly stable course of the disease. He had no history of prior admissions and he had his first-ever episode of sickle cell crisis lading in with acute chest syndrome, avascular necrosis of femur, and pulmonary embolism all at once. After an extensive review of the literature, we found this to be the first case report in the world where all these three complications of sickle cell disease developed simultaneously in a patient.

## Introduction

Sickle cell disease (SCD) is the name given to a group of inherited red blood cell disorders that affects hemoglobin, the body's oxygen-carrying protein. The presence of sickle hemoglobin (HbS) in red cells is the main characteristic of the disease and it usually has an autosomal recessive pattern of inheritance [1]. Sickle cell trait (SCT) or AS pattern refers to the individuals having a heterozygous state of the disease. Individuals showing homozygous traits for the mutation have SCD or SS pattern. The vaso-occlusive crisis also called sickle cell crisis is a painful complication of SCD in adolescents and adults which is relatively common [2]. Episodes of severe pain (crises) are the main reason for hospitalization of the SCD patients. The vaso-occlusive crisis, or sickle cell crisis, is initiated and sustained by interactions among sickle cells, endothelial cells, and plasma constituents. Vaso-occlusion is the culprit behind the clinical complications of sickle cell disease which includes pain syndromes predominantly. Other symptoms namely strokes, leg ulcers, spontaneous abortions, and renal insufficiency are also seen due to vaso-occlusion. 

Acute chest syndrome (ACS) is the term given for an acute pulmonary illness in a patient with sickle cell disease. ACS is defined by the presence of new pulmonary infiltrates on chest radiography and the presence of symptoms like fever and chest pain, along with cardinal signs and symptoms of pulmonary diseases namely tachypnea, cough, and dyspnea. Vaso-occlusion within the pulmonary vasculature of patients with sickle cell disease is thought to be the cause of ACS. This vaso-occlusion results in deoxygenation of hemoglobin and sickling of erythrocytes, which causes further occlusion leading to ischemia and injury to the vascular endothelium. ACS is characterized by quick progression and is the commonest cause of mortality in patients with sickle cell disease [3].

As sickle cell causes the red blood cells to become sticky and rigid, they can block blood flow in the small vessels supplying blood to the bone. Occlusion to these blood vessels leads to avascular necrosis of the hip or shoulder joint leading to loss of joint space [4]. At first, the patient complains of pain while putting weight on the affected joint however in the later stages patient develops pain even while resting. Another complication that may arise due to stasis of blood and hypercoagulable state is developing pulmonary embolism which might present with new-onset acute chest pain and differentiating it from acute chest syndrome might pose a challenge for the treating physicians [5].

## Case presentation

A 25-year-old male presented in the outpatient department with the chief complaint of severe back pain and hip pain for one day and fever for one day. The patient had a history of sickle cell disease SS pattern since childhood and was not on any medication. He had no history of similar episodes of bone pain and crisis-like episodes in the past. On physical examination, he was febrile with a temperature of 101.4 degrees Fahrenheit. He had severe pallor. His pulse rate was 130 beats per minute, respiratory rate was 20 breaths per minute, oxygen saturation level was 96% at room air, and blood pressure was 110/70 mm Hg, right arm supine position.

**Figure 1:**
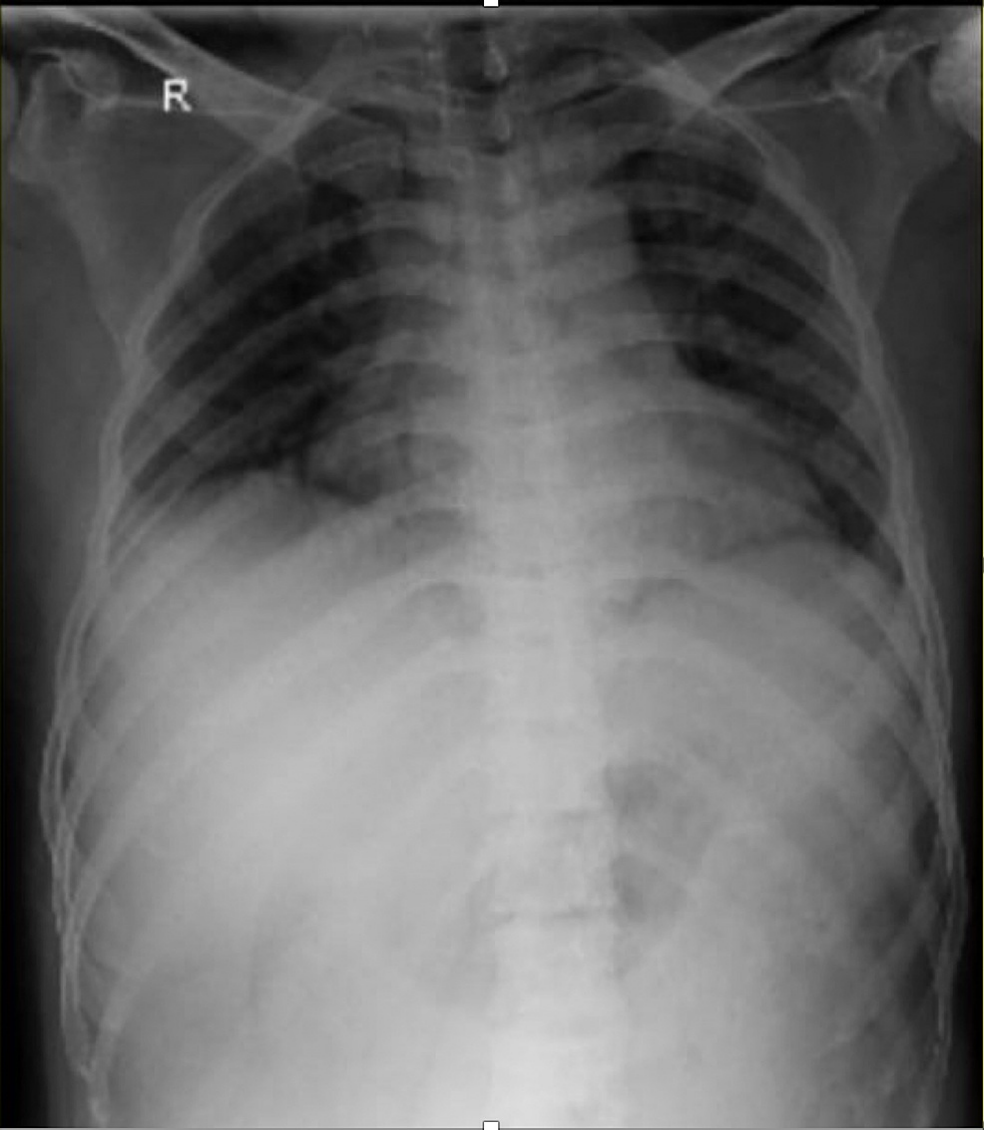
The image is showing a normal chest x-ray on admission

**Figure 2:**
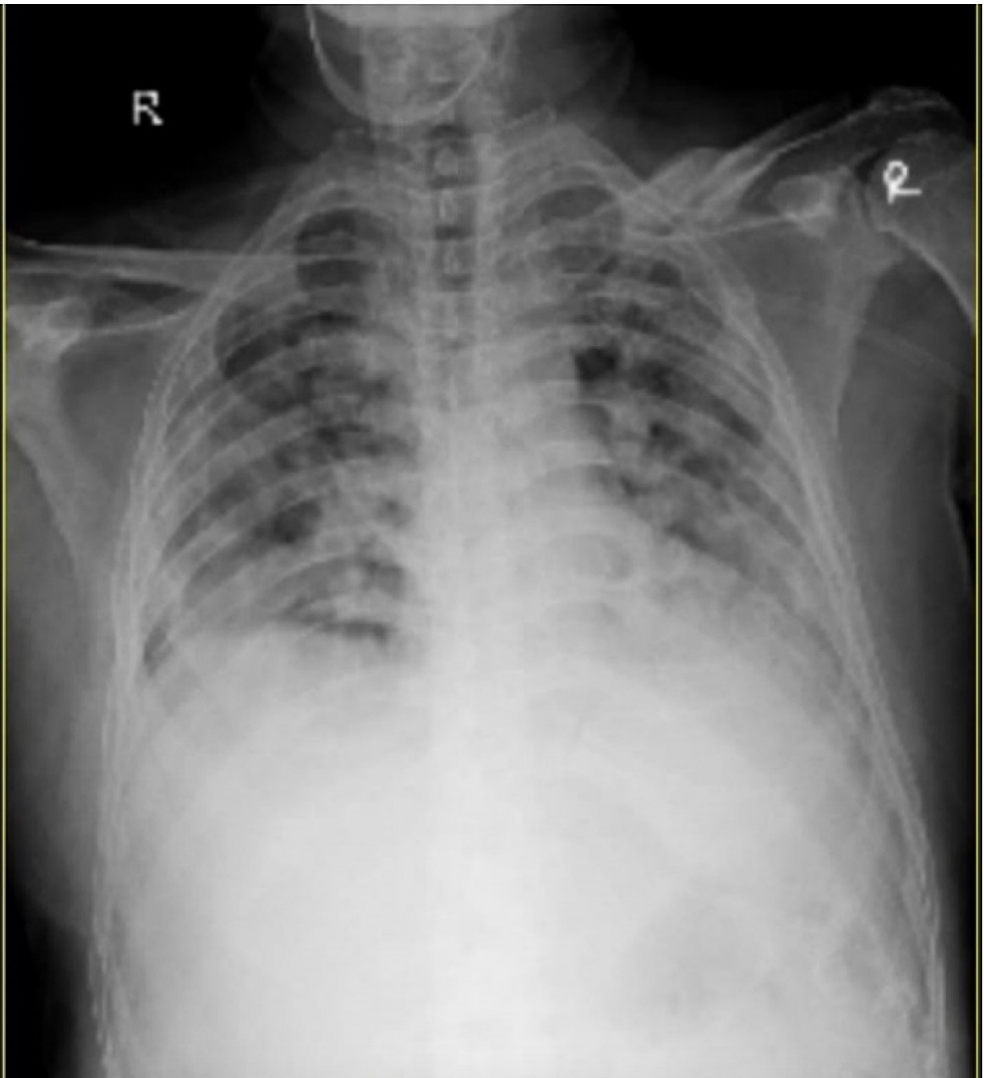
The image is showing the appearance of new infiltrates on the chest x-ray after the chest pain episode

**Figure 3:**
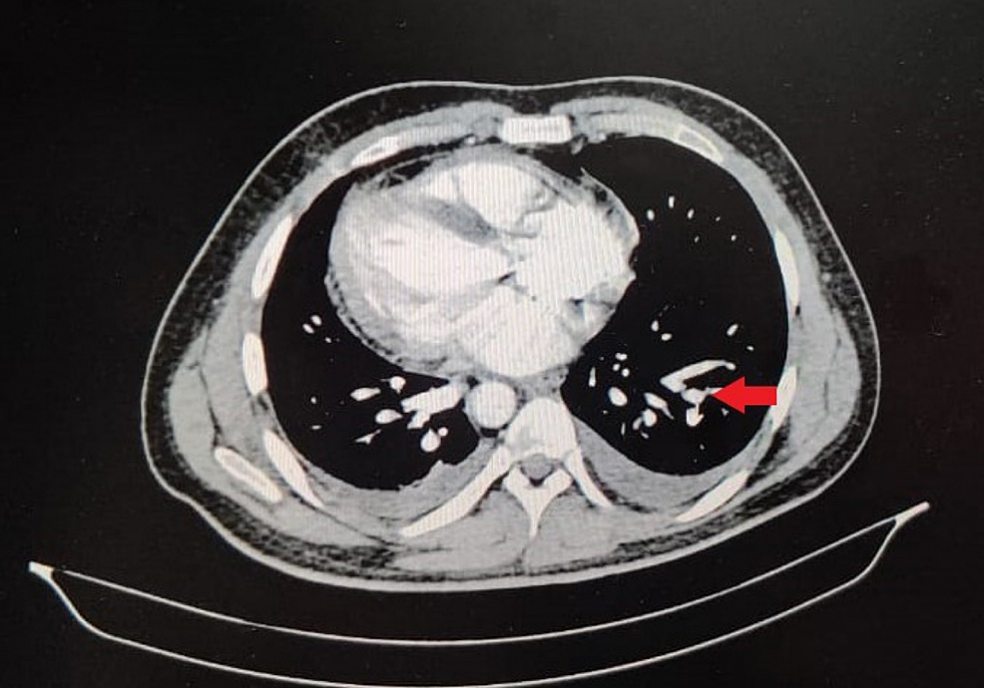
CT pulmonary angiography showing the presence of the sub-segmental embolus

**Figure 4:**
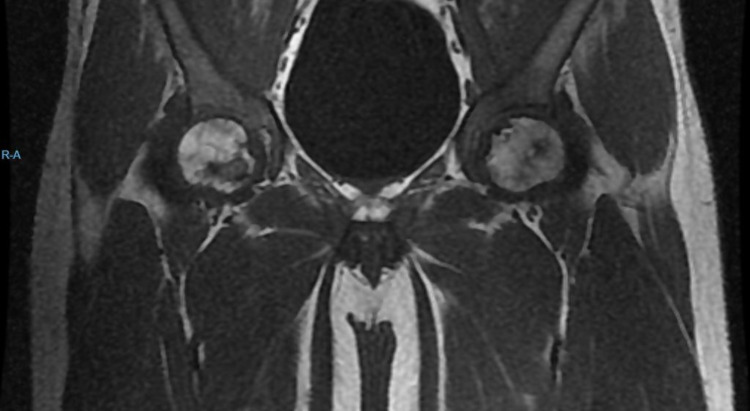
MRI of hip joint showing avascular necrosis of femur

Initial laboratory investigations showed anemia with hemoglobin 6.6 g%, white blood cell (WBC) count of 14,700 cells/mm^3^, out of which, 65% were granulocytes, platelet count was 3.27 lakhs/mm^3^, erythrocyte sedimentation rate (ESR) was 50 mm/h, and lactate dehydrogenase (LDH) levels were 605 U/L. Abdominal sonography reported grade I fatty liver of size 6.9 cm with hepatosplenomegaly and heterogenous echotexture of the spleen. The patient underwent two-dimensional echocardiography (2D ECHO), which reported mildly dilated left atrium and left ventricle. A chest radiograph done on admission showed no pulmonary infiltrates (Figure 1). The patient was started on IV antibiotics, analgesics, and IV fluids for the same. With the initial management, the patient felt symptomatically better. His complaints of pain were gradually reduced. Four days after admission, the patient started to experience complaints of chest pain which was non-radiating, had difficulty in breathing, and severe pain in the hip region. His saturation decreased to 85% on room air. He was put on oxygen support. Chest x-ray done revealed the presence of new infiltrates in the x-ray (Figure 2). CT pulmonary angiography revealed the presence of a small embolus in the subsegmental branch of the lower lobar pulmonary artery with the adjacent pulmonary infarct (Figure 3). MRI of hips was done which revealed avascular necrosis of the femur (Figure 4). Repeat blood investigations revealed a normal WBC count. Arterial blood gas (ABG) analysis was done that was suggestive of acidosis. With the above investigations, a diagnosis of inflammatory pulmonary disorder was taken into consideration. Given the normal WBC counts and the sudden desaturation and the appearance of new infiltrates on his x-ray, a diagnosis of acute chest syndrome was made. His antibiotics were stepped up accordingly. Appropriate pain medications were given. The patient was put on bilevel positive airway pressure (BIPAP) support. Plasma exchange was done to build up the optimum hemoglobin levels. Three days after presenting with the above symptoms, the patient started showing medical improvement. BIPAP support was gradually tapered, he was transferred to oxygen support, and eventually was maintaining saturation on room air.

After further hospitalization, his condition stabilized and all his chief complaints were treated. On the 12th day, he was discharged from the hospital and was called for follow-up. 

## Discussion

Acute chest syndrome is a life-threatening complication that is very preventable in patients who present with crisis episodes in sickle cell disease [6]. Adequate pain management, stepping up of antibiotics, maintaining optimum hemoglobin levels, oxygen support, and BIPAP remain the mainstay of treating a case of acute chest syndrome. Experimental treatments for ACS include inhaled nitric oxide, corticosteroid administration, and high-frequency oscillatory ventilation. ACS is the cause of 25% of mortality in sickle cell disease patients [7]. If untreated, patients could land up in a myriad of complications such as respiratory failure, neurological events namely altered mental status and seizures, pulmonary hemorrhage, cor pulmonale, overwhelming sepsis, and ultimately leading to death. The careful vigilance of the symptoms experienced by the patient, monitoring of the blood counts, serial chest x-rays, and maintenance of optimum hemoglobin levels are the preventive measures of a fatal outcome of the crisis thereby preventing acute chest syndromes.

Sickle cell disease is a hypercoagulable state where platelets tend to aggregate and this happens most commonly in the valve cusps of lower extremity veins [8]. A clot is formed which is then neutralized by fibrin. This process is repeated and multiple layers of fibrin and clots are formed resulting in the formation of venous thrombosis. Increase coagulability or venous stasis when increased will increase the risk of deep vein thrombosis and in turn development of pulmonary embolism [9]. A thrombus that breaks off and enters the pulmonary vasculature becomes pulmonary emboli and results in a potentially fatal condition for the patient. Stasis of blood in the blood vessels might occlude the blood supply to bones leading to necrosis of the bone [10]. Avascular necrosis of the femoral head is a relatively common complication in patients with sickle cell disease (SCD), and collapse of the femoral head occurs in 90% of the patients within five years of the diagnosis of osteonecrosis [11].

**Figure 5:**
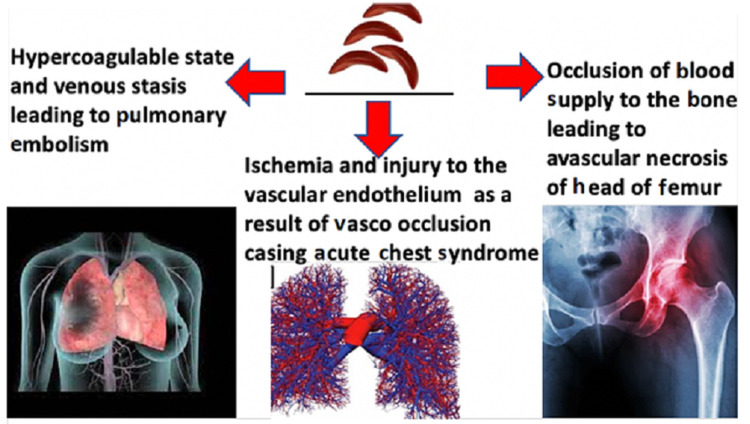
The image is showing pathophysiology of presentation of sickle cell disease in our case

The complications described above due to sickle cell disease are common after multiple episodes of crisis where the patient is admitted with acute onset pain due to repeated veno-occlusive episodes ultimately causing avascular necrosis of femur, ACS, and pulmonary embolism. However, it is interesting to note that our patient was stable with no history of hospitalization for sickle cell disease till the 25 years of his life, thereby denoting not even a single case of sickle cell crisis in his entire life. This case highlights a rare presentation of pulmonary embolism in a case of sickle cell disease who did not have any other signs of vaso-occlusive disease followed by the development of avascular necrosis of the femur which was suspected by mild pain in the hip joint. Our case later landed in another complication of sickle cell crisis with the development of acute chest syndrome which was unexpected (Figure 5). A sudden apocalypse in the form of pulmonary embolism, avascular necrosis of femur, and acute chest syndrome all occurring simultaneously in the first-ever crisis of sickle cell disease at 25 years of age make this case a rare one.

## Conclusions

A patient with asymptomatic SCD could suddenly develop life-threatening complications. Therefore, clinicians must maintain a high level of suspicion and astute observation. Sickle cell disease is very unique in its presentation where some patients remain asymptomatic for a larger part of their life and some patients present with repeated episodes of crisis. Therefore, we would like to conclude that, the first-ever presenting episode of a sickle cell crisis need not be a benign episode but can be life-threatening too. 
